# Synergistic Impact of Aerobic Exercise and Resveratrol on White Adipose Tissue Browning in Obese Rats: Mechanistic Exploration and Biological Insights

**DOI:** 10.3390/metabo15050331

**Published:** 2025-05-16

**Authors:** Yulong Hu, Yihan Wu, Chunlong Wang, Qiguan Jin, Xianghe Chen

**Affiliations:** College of Physical Education, Yangzhou University, Yangzhou 225009, China; 19210528299@163.com (Y.W.); 17626615371@163.com (C.W.); qgjin@yzu.edu.cn (Q.J.); xianghechen@yzu.edu.cn (X.C.)

**Keywords:** aerobic exercise, resveratrol, white fat browning, mitochondrial biogenesis

## Abstract

Obesity, marked by excessive white adipose tissue (WAT) accumulation, worsens metabolic disorders, and inducing WAT browning is a promising therapy. This study examined the synergistic effects of moderate-intensity aerobic training and resveratrol (RES) on WAT browning and its underlying mechanisms in obese male rats. **Methods:** Male Sprague Dawley rats were divided into a normal diet control group (*n* = 8) and a high-fat-diet modeling group (*n* = 32), with the rats in the latter group being further divided randomly in groups of eight into a high-fat group; a high-fat, exercise group; a high-fat, RES group; and a high-fat, exercise-combined-with-RES group. The rats in the exercise intervention groups underwent moderate-intensity aerobic treadmill exercise for one hour daily, six days a week, while those in the RES groups received a 50 mg/kg/d RES solution via gavage before exercise, once daily, six days a week. Both interventions lasted eight weeks. **Results:** The combined intervention synergistically suppressed weight gain and visceral fat accumulation. WAT browning was enhanced, evidenced by upregulated UCP1 and CIDEA expression. Mitochondrial biogenesis was activated via the SIRT1-PGC-1α-NRF-1-TFAM pathway, accompanied by elevated mitochondrial enzyme activity and improved lipid mobilization (reduced serum free fatty acids and triglycerides). **Conclusions:** The combination of aerobic exercise and RES promotes WAT browning and lipolysis by enhancing mitochondrial biogenesis and stimulating mitochondrial thermogenesis through the modulation of the SIRT1-PGC-1α-NRF-1-TFAM pathway.

## 1. Introduction

Obesity is an escalating global health crisis, driving up the prevalence of cardiovascular, digestive, and endocrine diseases. Adipose tissue is categorized into white adipose tissue (WAT) and brown adipose tissue (BAT), where WAT, an energy-storing tissue, comprises larger lipid droplets and fewer mitochondria, and BAT, an energy-consuming tissue, comprises smaller lipid droplets and more mitochondria. Uncoupling protein 1 (UCP1), a protein responsible for nonshivering thermogenesis, is highly expressed in brown adipocytes [1]. WAT browning denotes the morphological and functional changes occurring in white adipocytes under specific conditions, such as low temperature, exercise, or sympathetic activation, thus resulting in their transformation into beige adipocytes with brown adipocyte characteristics. Consequently, promoting WAT browning or enhancing BAT activity represents a promising therapeutic approach for combating obesity.

Some active plant ingredients, such as resveratrol (RES), canthaxanthin, and curcumin, have shown great potential in inducing WAT browning and BAT activation [2]. Notably, RES, a natural polyphenol found in grapes, red wine, peanuts, and some berries, has been shown to have anti-obesity effects [3]. Other studies have attributed this effect of RES to its ability to elevate the levels of peroxisome proliferator-activated receptor gamma coactivator-1 alpha (PGC-1α) and UCP1 in skeletal muscles and BAT, thus boosting mitochondrial function and energy expenditure [4]. However, the specific mechanism by which aerobic exercise combined with RES can brown fat has not been demonstrated, especially whether the combined intervention of the two enhances the SIRT1-PGC-1α signaling pathway through synergistic effects and thus promotes WAT browning. Clarifying this mechanism may provide a theoretical basis for the development of more effective obesity treatment strategies and promote the clinical application of combining natural ingredients with exercise.

Mitochondrial biogenesis, the process by which cells generate new mitochondria, is regulated by PGC-1α—which, upon activation by phosphorylation or deacetylation, first stimulates nuclear respiratory factor (NRF) 1 (NRF-1) and NRF-2 and then activates mitochondrial transcription factor A (TFAM) [5]. Moreover, silent information regulators (Sirtuins or SIRTs) play a role in WAT browning [6]; SIRT1-induced activation of PGC-1α is essential for the thermogenic program of WAT [7]. It has been found that exercise has a significant positive effect on fat activation and catabolism, and RES can promote mitochondrial biogenesis by activating the SIRT1-PGC-1α-NRF-1 pathway [8]. This study further investigates the effects of aerobic exercise combined with RES administration on WAT browning and the underlying mechanisms responsible for this browning process.

## 2. Materials and Methods

### 2.1. Main Reagents and Detection Methods

RES (purity ≥ 99%) was purchased from Shanghai McLean Biochemical Technology Co., Ltd. (Shanghai, China). The assay kits for triglycerides (TGs), total cholesterol (T-CHO), free fatty acid (FFA), cytochrome c oxidase (COX), and succinate dehydrogenase (SDH) were purchased from Nanjing Jiancheng Bioengineering Institute (Nanjing, China). TRIzol, the total ribonucleic acid (RNA) extraction reagent, was purchased from Ambion (Texas, TX, USA), the ReverTra AceTM qPCR RT Kit for reverse transcription was purchased from TOYOBO Co., Ltd. (Osaka, Japan), and the fluorescent dye-equipped quantitative real-time polymerase chain reaction (qPCR) kit, FastStart Universal SYBR Green Master (Rox), was purchased from Roche Diagnostics Corporation (Pleasanton, CA, USA).

### 2.2. Animals and Treatments

**Figure 1:**
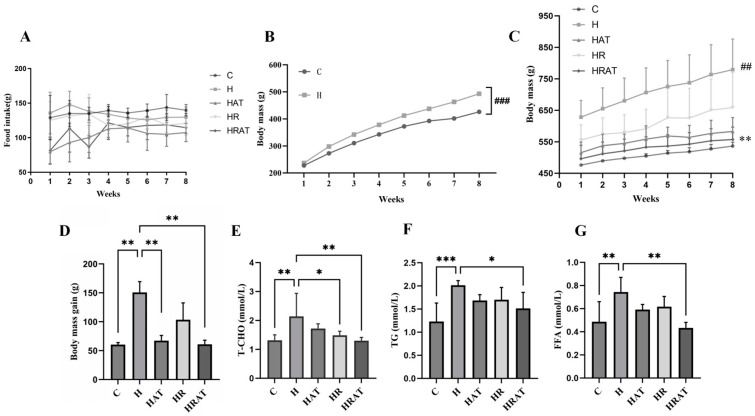
(**A**) Average daily food intake per week for each group of rats in the intervention phase. (**B**) Average weekly weight of rats in the control group and high-fat groups during the modeling phase. (**C**) Average weekly weight of rats in each group of rats in the intervention phase. “#” indicates significant differences compared to the C group. “*” indicates significant differences compared to the H group. (**D**) Average weight gain of rats in each group during the intervention phase. (**E**–**G**) Biochemical indices including values of total cholesterol (T-CHO), triglycerides (TGs), and free fatty acids (FFAs) for each group. Data are presented as the mean ± SEM. * *p* < 0.05; ** *p* < 0.01; *** *p* < 0.001. ## *p* < 0.01; ### *p* < 0.001.

In this study, fifty male Sprague Dawley (SD) rats, six to eight weeks old, were selected as experimental subjects and procured from the Jiangsu Laboratory Animal Center of Nanjing Medical University, Production License No. SYXK (Su) 2023-0081. They were housed in quartets in cages with an indoor temperature of 20–26 °C, a relative humidity of 40–70%, and a light/dark cycle of 12 h, and they were given free access to food and water. Following one week of acclimatization with standard feed (Synergy: 1010088), we began an 8-week period to establish the obesity model. During this modeling phase (as shown in Figure 1B), the rats were randomly allocated into a control group (*n* = 8) and a high-fat group (*n* = 42). The control group continued to receive standard chow, while the high-fat group was fed a high-fat diet (Synergy: XTHF45-1), in which 47% of the calories came from fats, 20% from proteins, and 33% from carbohydrates. After 8 weeks of obesity modeling, rats that exhibited a body weight gain and Lee’s Index scores 20% higher than the mean values of the normal-diet control (C) group, with Lee’s Index scores ≥ 310, were identified as obese [9]. The thirty-two obese rats meeting these criteria were further randomly divided into four groups: the high-fat-diet (H) group, the high-fat, exercise (HAT) group; the high-fat, RES (HR) group; and the high-fat, exercise-combined-with-RES (HRAT) group, with eight rats in each group. The intervention phase was initiated in week 10; 50 mg/kg/d of RES was administered to the HR and HRAT groups in solution form at a concentration of 25 mg/mL by gavage, and an equal volume of 2% dimethyl sulfoxide (DMSO) solution was given to the H and HAT groups. The administration was carried out six times per week for eight weeks. The body weight and food intake of the rats were recorded on a weekly basis. The rats were euthanized 24 h after the end of the last intervention, and adipose tissue was dissected out and weighed. Serum, inguinal WAT (iWAT), and perirenal WAT (vWAT) were collected and stored at −80 °C. This study was reviewed and approved by the Laboratory Animal Welfare Ethics Committee of Yangzhou University (Grant No. 202407037).

### 2.3. Exercise Training

Rats in the HAT and HRAT groups underwent exercise intervention one hour after gavage, starting from week 10. An incremental load running test was conducted on both groups of rats at the beginning of the exercise intervention to ascertain the maximum running speed. During the test, the treadmill slope was set to 0°; the initial running speed was set to 10 m/min and was gradually increased in 2 m/min increments until the rats reached their endurance limit and could no longer continue running after several attempts. The speed of the penultimate stage was considered as the maximum speed. The exercise regimen consisted of a 10 min warm-up phase (the speed of the treadmill was 10 m/min), a 40 min moderate-intensity aerobic phase (where a speed 60–70% of the maximal speed was maintained here), and a 10 min recovery phase (the speed was 10 m/min). The training sessions were conducted at 8:00 p.m. 6 times per week for 8 weeks with an elevation angle of 0°. Rats in groups C, H, and HR did not receive exercise intervention.

### 2.4. Quantitative Real-Time PCR

The total RNA from iWAT and vWAT was extracted using TRIzol reagent and subsequently quantified using an ultramicro spectrophotometer and reverse-transcribed into complementary deoxyribonucleic acid (cDNA). qPCR was performed using the SYBR Green Master mix, and the fold change in target gene expression was calculated using the relative quantification method (2^−ΔΔCT^). The primer sequences for the target genes are presented in Table 1.

### 2.5. Western Blotting

The protein samples were subjected to 10% sodium dodecyl sulfate polyacrylamide gel electrophoresis (SDS-PAGE). The polyvinylidene difluoride (PVDF) membrane was activated and equilibrated and then positioned within a rotary folder following a specific sandwich configuration for 10–15 min. The transferred proteins were detected using the immunological method, which entails rinsing and containment, followed by incubation first with a primary antibody (the uncoupling protein 1 [UCP1] primary antibody [1:1000, ABclonal, Woburn, MA，USA]) and then with a secondary antibody (HRP-conjugated Goat anti-Rabbit IgG (H + L) [1:10,000, ABclonal]), culminating in electrochemiluminescence (ECL) imaging; the imaging results were scanned in grayscale.

### 2.6. Statistical Analyses

Statistical analyses were performed using GraphPad Prism 10.1.2. Independent sample *t*-tests were used for comparison between groups C and H, and a two-way analysis of variance (ANOVA) followed by Tukey’s post hoc test was employed for drawing comparisons between groups H, HAT, HR, and HRAT. *p* < 0.05 was considered statistically different.

## 3. Results

### 3.1. Effects of Aerobic Exercise Combined with RES on Body Weight and Fat Metabolism in Rats

The weekly dietary regimens of the rats during the intervention phase are presented in Figure 1A, and no substantial differences were observed in energy intake between the groups. After 8 weeks of high-fat feeding, the rats with Lee’s Index scores ≥ 310 and weight gain and Lee’s Index scores that were 20% higher than the mean value of group C were classified as obese; the weight of the rats in the high-fat group was significantly higher than that of the rats in the control group (*p* < 0.001, Figure 1B). After eight weeks of exercise and pharmacological intervention (Figure 1C,D), the rats in the HRAT group, having received both exercise and pharmacological interventions, exhibited significantly lower weights (*p* < 0.01) and weight gain rates (*p* < 0.001) than all the other high-fat-diet rats. In addition, the high-fat diet substantially increased the levels of T-CHO, TG, and FFA in the plasma of the rats, but supplementation with RES or exercise combined with RES resulted in a certain degree of improvement in the lipid levels (Figure 1E–G).

### 3.2. Aerobic Exercise Combined with RES Inhibits the Increase in Adipose Tissue Content and Enhances Lipolysis in Rats Fed a High-Fat Diet

**Figure 2:**
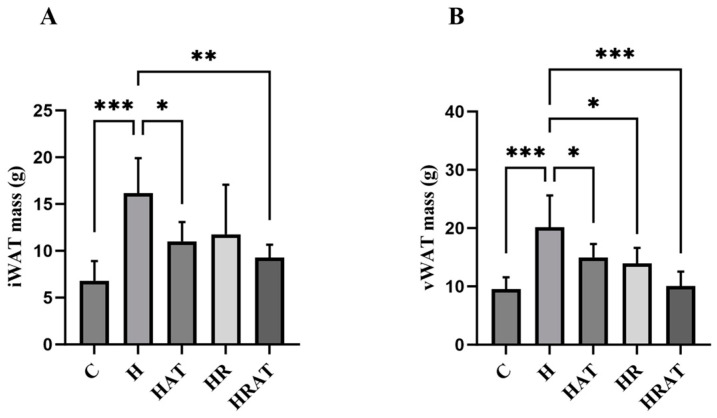
(**A**,**B**) iWAT and vWAT content, respectively. Data are presented as the mean ± SEM. * *p* < 0.05; ** *p* < 0.01; *** *p* < 0.001.

Safahani et al. [10] demonstrated in an experiment involving dietary intervention in adult male C57BL/6J mice that RES, a naturally occurring polyphenol, reduces adipose tissue content. Despite adipose tissue being an appealing target for studying the action of RES, limited studies to date have explored the application of RES to this tissue [11], and even fewer have examined its use with aerobic exercise. Following the dissection of the rats, their iWAT (Figure 2A) and vWAT (Figure 2B) were isolated and weighed. The results showed that both aerobic exercise and RES inhibited the increase in iWAT and vWAT content, and the effect was better when they were combined. In addition, this study also found that aerobic exercise and RES controlled vWAT content better than they did iWAT. This could be attributed to visceral fat being more sensitive to the increase in adipocyte volume induced by high-fat feeding [12].

**Figure 3:**
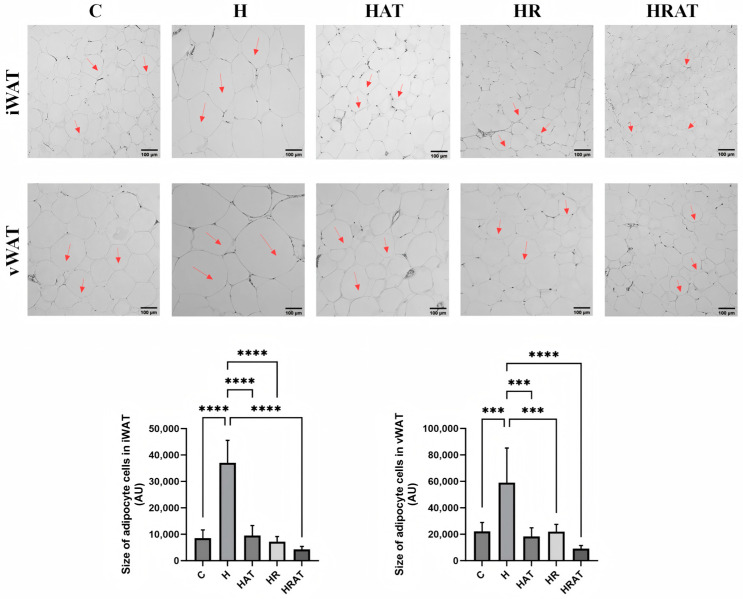
Hematoxylin and eosin staining and size of adipocyte cells of iWAT and vWAT (scale bar = 100 µm); magnification = 100×. The red arrow indicates the change in droplet size. Data are presented as the mean ± SEM. *** *p* < 0.001; **** *p* < 0.0001.

To enhance the clarity of visualizing adipocyte size, hematoxylin and eosin (HE) staining was conducted on inguinal adipocytes and perirenal adipocytes of the five groups. As illustrated in Figure 3, the adipocytes in rats of the HAT, HR, and HRAT groups were smaller than those in rats of the H group, with rats of the HRAT group having the smallest adipocytes. This indicates that aerobic exercise combined with RES reduces the accumulation of lipids in rats on a high-fat diet.

### 3.3. Aerobic Exercise Combined with RES Triggers the Browning of WAT and Enhances Thermogenesis in BAT

**Figure 4:**
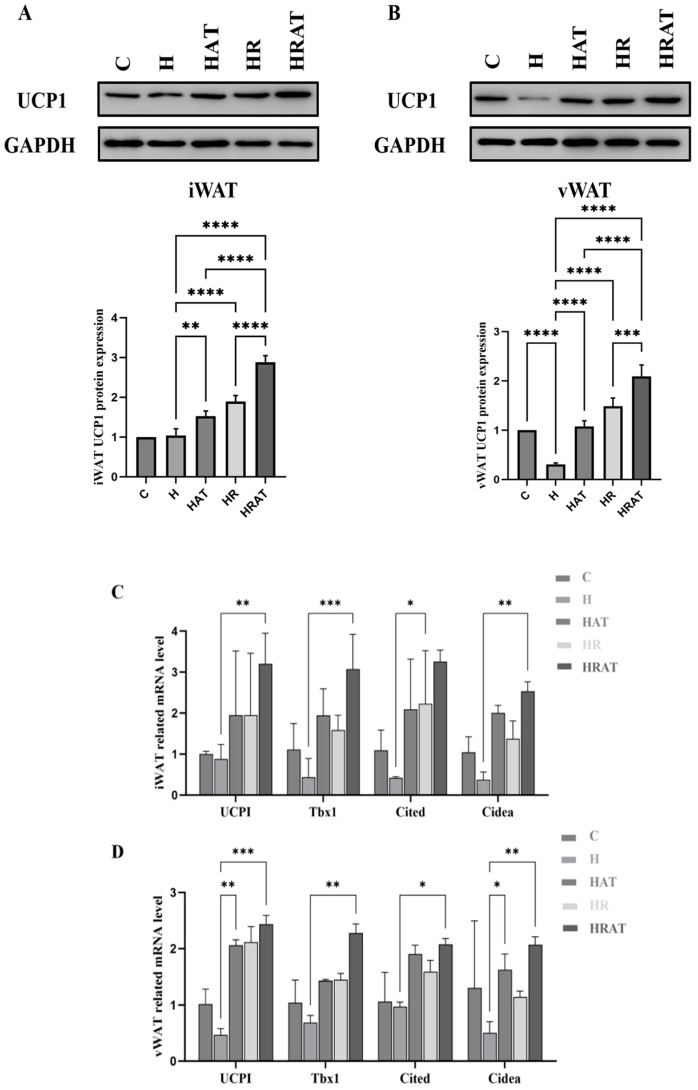
(**A**,**B**) Protein expression levels of UCP1 in iWAT and vWAT, respectively. (**C**,**D**) Expression of mRNA levels of brown-specific genes (UCP1, Cidea) and beige-specific genes (Cited1, Tbx1) in iWAT and vWAT, respectively. Data are presented as the mean ± SEM. * *p* < 0.05; ** *p* < 0.01; *** *p* < 0.001; **** *p* < 0.0001.

BAT, recognized as a thermoregulatory tissue that promotes energy expenditure, has been widely studied for its potential role in combating obesity [13]. Furthermore, UCP1 expression serves as a functional biomarker for evaluating adipocyte thermogenic potential and distinguishing BAT from WAT [14]. Western blotting (WB) of iWAT (Figure 4A) and vWAT (Figure 4B) lysates showed that UCP1 expression in the HAT, HR, and HRAT groups was significantly higher than that in the H group, with the HRAT group exhibiting the highest UCP1 expression of all. The results of reverse transcription PCR (RT-PCR) experiments (Figure 4C,D) for UCP1 expression were consistent with those of WB. In addition, the mRNA levels of cell-death-inducing DNA fragmentation factor-like effector A (CIDEA), Cbp/p300-interacting transactivator (CITED), and T-box transcription factor 1 (Tbx1), proteins highly expressed in brown or beige adipocytes, were also higher in the HAT and HR groups than in the H group, with the highest levels being detected in the HRAT group. In conclusion, the combination of aerobic exercise and RES can induce the browning of WAT and promote thermogenesis in BAT.

### 3.4. Aerobic Exercise Combined with RES Promotes White Adipose Tissue Mitochondrial Biogenesis Through a Multidimensional Mechanism

**Figure 5:**
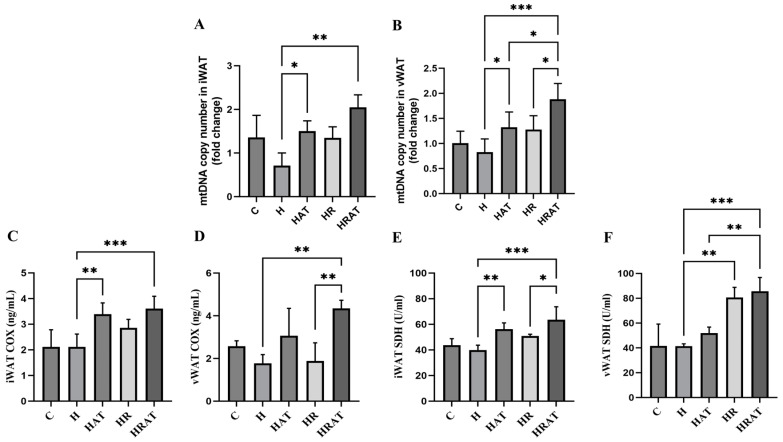
(**A**,**B**) mtDNA copy number in iWAT and vWAT. (**C**,**D**) COX content in iWAT and vWAT, respectively. (**E**,**F**) SDH content in iWAT and vWAT, respectively. Data are presented as the mean ± SEM. * *p* < 0.05; ** *p* < 0.01; *** *p* < 0.001.

In this study, we confirmed that aerobic exercise combined with RES intervention significantly promoted mitochondrial biogenesis during white adipose tissue (WAT) browning by multidimensional assessment. To comprehensively assess the functional status of mitochondria, we used the following experimental methods: (1) mtDNA (mitochondrial DNA) copy number was detected by real-time fluorescence quantitative PCR (RT-PCR) (Figure 5A,B), which showed that the mtDNA content was significantly increased in the combined intervention group compared with the H group (*p* < 0.01), confirming an active replication of the mitochondrial genome; and (2) enzyme activity assays showed that both complex IV (COX) activity (Figure 5C,D) and complex II (SDH) activity (Figure 5E,F) were significantly enhanced. Notably, the elevated SDH activity—a nuclear DNA-encoded mitochondrial marker—further corroborated that this biogenesis process operates independently of mtDNA damage repair. Together, these results indicate that the synergistic effect of aerobic exercise and RES promotes mitochondrial biogenesis at both the genetic level (mtDNA content) and functional level (enzyme activity).

### 3.5. Aerobic Exercise Combined with RES Promotes Mitochondrial Biogenesis Through the SIRT1-PGC-1α-NRF-1-TFAM Pathway

**Figure 6:**
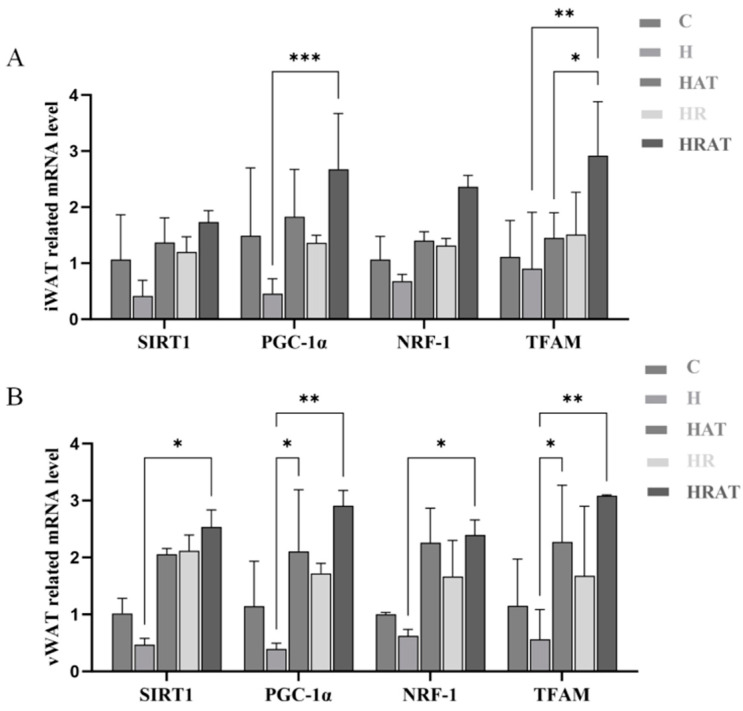
(**A**,**B**) mRNA expression levels of genes related to mitochondrial biogenesis (SIRT1, PGC-1α, NRF-1, TFAM) expressed in iWAT and vWAT, respectively. Data are presented as the mean ± SEM. * *p* < 0.05; ** *p* < 0.01; *** *p* < 0.001.

One of the most extensively investigated mechanisms of the action of RES is boosting energy expenditure by regulating protein targets in central energy pathways and signaling, particularly by inducing mitochondrial biogenesis [11]. RES promotes mitochondrial biogenesis and adipocyte thermogenesis by activating the protein deacetylase SIRT1, which subsequently results in the deacetylation and activation of PGC-1α [2,15]. As a key marker of thermogenesis, PGC-1α can interact with NRF-1 to regulate TFAM, thereby stimulating mitochondrial biogenesis and the expression of proteins involved in mitochondrial respiration signaling pathways [6]. As illustrated in Figure 6, both aerobic exercise and RES supplementation increased the expression of SIRT1, PGC-1α, NRF-1, and TFAM, and the synergistic effect was more significant when aerobic exercise was facilitated in conjunction with RES administration, confirming that RES supplementation and aerobic exercise collectively activate the SIRT1-PGC-1α-NRF-1-TFAM pathway and promote mitochondrial biogenesis.

## 4. Discussion

Obesity is recognized as a chronic metabolic disorder characterized by dysregulated balance between WAT and BAT that results in excessive accumulation of WAT in the body [2]. Mammals possess three distinct adipocyte populations: white, brown, and beige adipocytes. Given the remarkable energy-dissipating capacity of brown fat, therapeutic strategies promoting white-to-brown fat transdifferentiation have emerged as promising anti-obesity approaches.

One recent review [16] writes that over the past 20 years, there has been increasing clinical evidence of the health benefits of resveratrol. RES has demonstrated efficacy in reducing body weight and adipocyte hypertrophy in obese subjects [17,18]. The anti-obesity properties of RES may be attributed to its ability to inhibit adipogenesis [19], increase mitochondrial capacity and stimulate fat oxidation [20], stimulate WAT browning [7], upregulate UCP1, and enhance thermogenesis [7,21]. Using a rodent model, Stanford et al. [22] established that exercise training also promoted iWAT and vWAT mitochondrial biogenesis, increased the number of brown adipocytes, enhanced UCP1 expression, and induced the browning of WAT. However, few researchers have examined the effects of aerobic exercise in combination with RES supplementation. This study therefore investigated the combinatorial impact of aerobic exercise and RES on WAT browning in obese rats and explored their mechanism of action.

This study found that the two interventions, aerobic exercise and RES, showed significant synergistic effects in suppressing body weight gain (Figure 1C,D). Notably, this effect was independent of caloric intake, as there was no substantial difference in diet portions among the five groups (Figure 1A), suggesting enhanced energy expenditure as the primary mechanism. These findings align with Kang et al. [23], who reported RES-mediated inhibition of adipocyte proliferation accompanied by reduced glycerol-3-phosphate dehydrogenase (GPDH) and TG levels, suggesting that RES has lipolytic and thermogenic properties. This was consistent with the results of the present study, where T-CHO, TG, and FFA levels were downregulated in combined intervention with aerobic exercise and RES (Figure 1E–G).

Morphometric analyses revealed substantial reductions in adipocyte size within both iWAT and vWAT following exercise and RES treatments (Figure 2 and Figure 3). This observation corroborates previous work by Gollisch et al. [12], demonstrating that exercise-induced improvements in insulin sensitivity correlate with morphological remodeling of adipose tissue, particularly in visceral depots. In the present study, more pronounced changes in adipocytes were likewise observed in vWAT (Figure 2 and Figure 3).

Moreover, the browning process was further characterized by significant upregulation of UCP1 expression (Figure 4A,B), along with increased mRNA levels of brown/beige adipocyte markers (UCP1, CIDEA, CITED1, and Tbx1) in both iWAT and vWAT (Figure 4C,D). Beige adipocytes, sharing morphological and functional characteristics with classical brown adipocytes (including multilocular lipid droplets, abundant mitochondria, and high UCP1 expression) [24,25], were particularly induced by the combined intervention.

Mitochondria, ubiquitous organelles in eukaryotic cells, serve as central hubs for both catabolic and biosynthetic processes, facilitating diverse metabolic reactions such as fatty acid synthesis, the tricarboxylic acid (TCA) cycle, and acetate oxidation, while also generating signaling molecules that regulate cellular metabolism [26]. A PCR assay of the mtDNA copy number showed (Figure 5A,B) that the mitochondrial genome content was significantly increased in the HRAT group compared to the H group (*p* < 0.01). Moreover, the increased activities of COX and SDH (Figure 5C–F) further validated the synergistic effect of this combined intervention on mitochondrial biogenesis. Mechanistically, these effects appear mediated through the SIRT1-PGC-1α-NRF1-TFAM axis. This pathway aligns with established mechanisms, whereby (1) PGC-1α activation stimulates mitochondrial protein and DNA synthesis [5], (2) RES activates SIRT1-PGC-1α signaling [8], and (3) caloric restriction (mimicked by RES) can activate SIRT1-mediated fat mobilization [27].

Supporting this model, Andrade et al. [28] reported that RES-induced metabolic improvements correlate with increased UCP1 and SIRT1 expression, while SIRT1 knockdown studies confirm its essential role in BAT function [29]. It is worth noting that the activation of SIRT1 by RES not only restores UCP1 levels that were impacted by inflammation [30] but also deacetylates PGC-1α [2,31], subsequently activating downstream effectors NRF-1 and TFAM [32,33]. MtDNA quantification (Figure 5A,B) provides the first direct evidence that exercise–RES combination therapy amplifies the mitochondrial genomic reservoir through TFAM-dependent mechanisms.

This study reveals, for the first time, that aerobic exercise and RES have a significant synergistic effect in promoting the browning of white adipose tissue. Mechanistic studies showed that aerobic exercise and RES together significantly enhanced the transcriptional activities of NRF-1 and TFAM by activating SIRT1-mediated deacetylation of PGC-1α (Figure 6), thereby effectively promoting mitochondrial biogenesis.

## 5. Conclusions

In conclusion, the current investigation has provided further validation that aerobic exercise combined with resveratrol significantly inhibits the increase in body weight and adipose tissue content caused by a high-fat diet, and it promoted lipolysis in vivo by increasing mitochondrial biogenesis and white fat browning. This effect may be mediated through the signaling pathway SIRT1-PGC-1α-NRF-1-TFAM. It is anticipated that the findings from this study will offer novel insights and pave the way for potential clinical applications of aerobic exercise in conjunction with resveratrol in human populations.

## Figures and Tables

**Table 1:** Primers for real-time PCR.

Gene Name	Forward Sequences (5′-3′)	Reverse Sequences (5′-3′)
GAPDH	5′-TGCCACTCAGAAGACTGTGG-3′	5′-TTCAGCTCTGGGATGACCTT-3′
UCP1	5′-GGGCTGATTCCTTTTGGTCTCT-3′	5′-GGGTTGCACTTCGGAAGTTGT-3′
Cidea	5′-TGTTAAGGAGTCTGCTGCGGTTC-3′	5′-ATGGCTGCTCTTCTGTGTCACC-3′
Cited	5′-AAGCCAACCAGGAGAGGATGAG-3′	5′-GGGCACCAGCAGGAGAGAC-3′
Tbx1	5′-GGCAGGCAGACGAATGTTCCC-3′	5′-CAGCCACCAGCCAGGAGGAG-3′
SIRT1	5′-ATTTATGCTCGCCTTGCTGTG-3′	5′-CAGAGCTGCTCATGAATGCTG-3′
PGC-1α	5′-TATTCATTGTTCGATGTGTCGC-3′	5′-TGTCTGTAGTGGCTTGATTCAT-3′
NRF-1	5′-TCTGCTGTGGCTGATGGAGAGG-3′	5′-GATGCTTGCGTCGTCTGGATGG-3′
TFAM	5′-GCAGAAACGCCTAAAGAAGAAAGC-3′	5′-ACTCATCCTTAGCCTCCTGGAAG-3′
MT-ND1	5′-CAGGACCATTCGCCCTATTCT-3′	5′-ATAGGAGGTGCATTAGTTGGTCAT-3′

## Data Availability

Publicly available datasets were analyzed in this study. The original contributions presented in the study are included in the article. Further inquiries can be directed to the corresponding author.

## Abbreviations

The following abbreviations are used in this manuscript:

RES	Resveratrol
WAT	White adipose tissue
BAT	Brown adipose tissue
iWAT	Inguinal adipose tissue
vWAT	Visceral adipose tissue
T-CHO	Total cholesterol
TG	Triglyceride
FFA	Free fatty acid
COX	Cytochrome c Oxidase
SDH	Succinate dehydrogenase
mtDNA	Mitochondrial DNA
UCP1	Uncoupling protein 1
Cidea	Cell Death-Inducing DNA Fragmentation Factor Alpha-like Effector A
Tbx1	T-Box Transcription Factor 1
SIRT1	Sirtuin 1
PGC-1α	Peroxisome proliferator-activated receptor gamma coactivator-1 alpha
NRF-1	Nuclear Respiratory Factor 1
TFAM	Mitochondrial Transcription Factor A
